# Mulberry Leaf-Derived Bioactive Constituents on Diabetes: Structure, Extraction, Quality Analysis, and Hypoglycemic Mechanisms

**DOI:** 10.3390/molecules31020367

**Published:** 2026-01-20

**Authors:** Siyue Zhou, Yidong Xu, Yehao Lin, Junyu Liu, Min Zhang, Joseph Buhagiar, Haixia Chen

**Affiliations:** 1 Tianjin Key Laboratory for Modern Drug Delivery & High-Efficiency, School of Pharmaceutical Science and Technology, Faculty of Medicine, Tianjin University, Tianjin 300072, China; zhou_624@tju.edu.cn (S.Z.); mita@tju.edu.cn (Y.X.); junyuliu@tju.edu.cn (J.L.); 2 Nutritious and Healthy Food Sino-Thailand Joint Research Center, College of Food Science and Biological Engineering, Tianjin Agricultural University, Tianjin 300384, China; 3 State Key Laboratory of Nutrition and Safety, Tianjin University of Science & Technology, Tianjin 300457, China; 4 Faculty of Science, University of Malta, MSD 2080 Msida, Malta; joseph.buhagiar@um.edu.mt

**Keywords:** diabetes mellitus, mulberry leaf, *Morus alba* L., 1-deoxynojirimycin, quality analysis, hypoglycemic mechanism, alpha-glucosidase

## Abstract

(1) Background: Diabetes mellitus is a chronic metabolic disease with a rising global prevalence. Mulberry leaf (ML), a traditional medicinal and edible plant, possesses notable hypoglycemic effects and has a long history of usage. This review aims to systematically consolidate the research progress on the hypoglycemic constituents derived from ML, including their chemical structure, extraction methods, quality analysis techniques, and hypoglycemic mechanisms. (2) Methods: Adhering to the Preferred Reporting Items for Systematic Reviews (PRISMA 2020) guidelines, a comprehensive literature search was conducted using Web of Science and PubMed databases to find relevant studies published between 2015 and 2025. (3) Results: This review evaluates both conventional and modern techniques such as water extraction, ultrasound-assisted extraction (UAE), microwave-assisted extraction (MAE), and enzyme-assisted extraction (EAE), highlighting their advantages and limitations when applied on ML. Additionally, this review examines the analytical techniques applied in the quality control of ML and its constituents. This is complemented by a summary of hypoglycemic mechanisms, focusing on the inhibition of oxidative stress, amelioration of insulin resistance, regulation of related enzyme activity, and modulation of gut microbiota. (4) Conclusions: ML demonstrates considerable potential for treating diabetes. However, further studies are needed for new drug discovery based on new ML-derived bioactive constituents, highly efficient extraction methods, quality analysis techniques, and underlying mechanisms.

## 1. Introduction

Diabetes mellitus is a chronic metabolic disorder that has become a global public health concern due to its increasing prevalence, caused by socio-economic development and changes in lifestyle [1]. According to data from the 11th edition of the International Diabetes Federation (IDF) Diabetes Atlas, the global population of individuals with diabetes has reached 643 million, and the number is projected to increase to 853 million by the year 2050, representing approximately one-eighth of the world’s population. About 90% of diabetes are classified as type 2 diabetes mellitus (T2DM), which is characterized by insufficient insulin secretion and is closely associated with β-cell dysfunction and insulin resistance [2]. At present, active lifestyle interventions and pharmacological treatments are recognized as effective approaches for treating T2DM. Although most clinically used drugs effectively lower blood glucose levels, they also have side effects, including the risk of hypoglycemia, weight gain, drug resistance, and increased burden on the liver and kidneys [3]. Therefore, there is an urgent need to develop novel therapies for T2DM that are safe and effective. In recent years, natural products have become a research focus due to their high efficacy and low side effects. For instance, Sangzhi Total Alkaloids Tablets were officially approved as a new anti-diabetic drug in 2020, underscoring the significant research value of natural products in the field of diabetes treatment [4].

*Morus alba* L., a rapidly growing deciduous plant, is widely distributed across temperate, subtropical, tropical, and arid regions [5]. Each part of *Morus alba* L., including the leaf, fruit, twig, and root bark, has various medicinal properties [6]. Among them, the leaf of *Morus alba* L. is a well-known medicinal and edible homologous traditional Chinese medicine, valued for its abundant resources and high utilization potential. In traditional Chinese medicine, ML was first recorded in Shennong’s Herbal, the earliest Chinese pharmacopeia, and has been used to treat diabetes for a long time [7]. In modern research, ML extract has demonstrated properties that reduce inflammation and oxidative stress, protect pancreatic β-cells, and regulate glucose and lipid metabolism, suggesting its potential as a promising therapeutic option for diabetes [8]. Moreover, numerous studies have shown that flavonoids, alkaloids, polysaccharides, and phenolic acids are the primary molecular basis of ML’s capacity to lower blood sugar [9]. For instance, 1-deoxynojirimycin (1-DNJ) is a representative alkaloid found in ML and is a famous naturally occurring hypoglycemic compound. 1-DNJ was initially derived from nojirimycin in 1966 and subsequently isolated and characterized from the root bark of *Morus alba* ten years later. Further investigations revealed the presence of 1-DNJ in additional parts of the mulberry plant [10]. The hypoglycemic properties of ML are closely related to the distinctive structure of its active constituents, the selection of extraction methods, and the development of quality analysis techniques. Research on the hypoglycemic mechanisms of ML provides a basis for further investigation and the development of natural antidiabetic medications and medicinal ML resources. Although a lot of the existing literature explores the effects of ML in treating diabetes and its complications, few articles have systematically reviewed the extraction methods, quality analysis, and hypoglycemic mechanisms of the active components in ML.

This review summarizes research progress on the anti-diabetic effects of ML from 2015 to 2025, covering the extraction of hypoglycemic constituents, quality analysis, and hypoglycemic mechanisms. This review will provide inspiration for researchers in the development of new hypoglycemic drugs and functional foods derived from ML.

## 2. Results

### 2.1. Database Search Results

**Figure 1:**
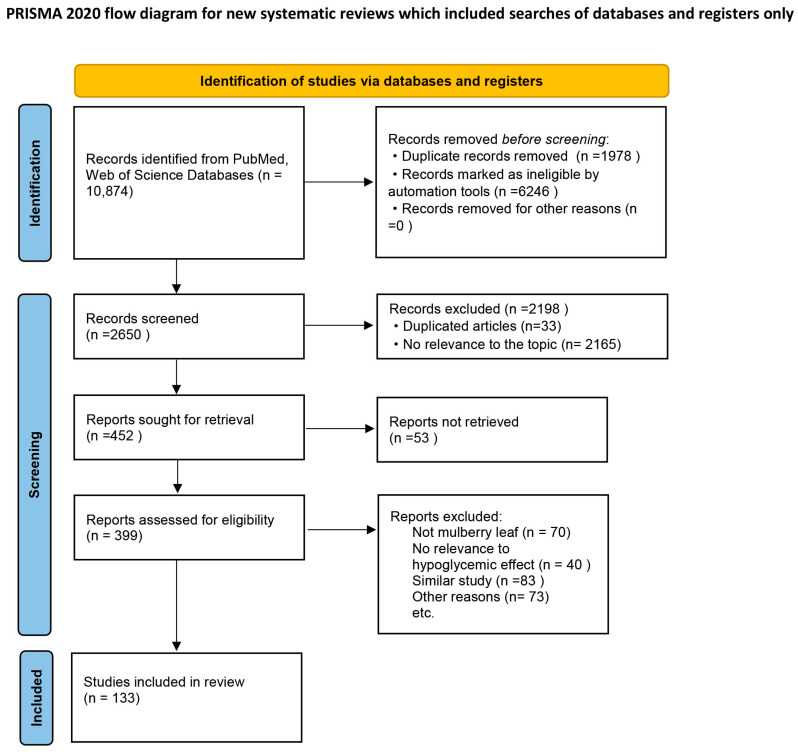
The flowchart of the selection of literature and reports based on PRISMA.

The literature for this review was searched from two different databases: PubMed and Web of Science. The search and selection processes are illustrated in the PRISMA flow diagram (Figure 1). From the initial database search, a total of 10,874 records were identified: 1144 records from PubMed and 9730 records from Web of Science. Of these, 6246 ineligible records, such as patents, letters, meetings, news, reports, etc., were excluded using an automated tool. After removing duplicates, 2650 studies were ultimately retained. Further screening based on the title and abstract of articles resulted in 452 articles being deemed highly relevant to the scope of this review. However, 53 of these were excluded because their full texts could not be obtained. To determine whether the remaining 399 articles were compliant with the inclusion criteria, their full texts were thoroughly reviewed and evaluated. Articles were removed after full-text screening if they did not pertain to ML, were not relevant to the hypoglycemic effect, or were similar studies. At the end of the screening flow, a total of 133 articles were included.

### 2.2. Hypoglycemic Constituents of ML

**Figure 2:**
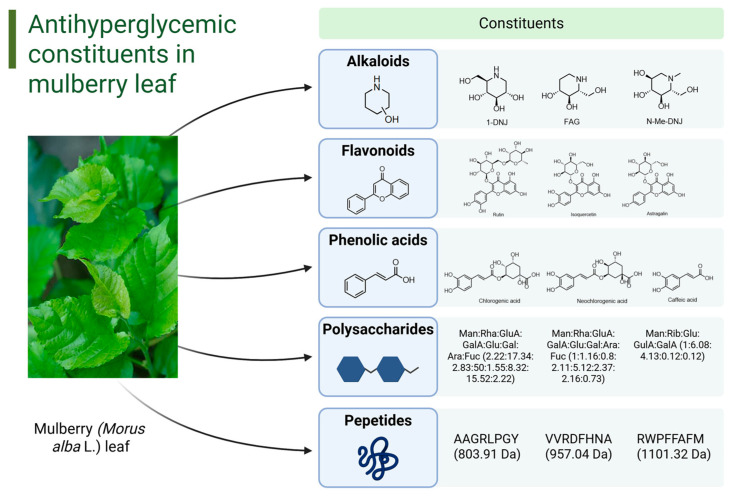
The main categories of antihyperglycemic constituents in ML along with the structures of their representative compounds (created in https://BioRender.com).

ML contains a variety of phytochemicals with hypoglycemic activity, such as alkaloids, flavonoids, phenolic acids, polysaccharides, and peptides. Figure 2 illustrates the main categories of antihyperglycemic constituents in ML along with the structures of their representative compounds. The hypoglycemic compounds identified in ML, as supported by the existing literature, are listed in Table 1.

#### 2.2.1. Mulberry Leaf Alkaloids (MLA)

Alkaloids are widely recognized as one of the most important hypoglycemic constituents in ML. In 1994, the presence of seven alkaloids in ML, including 1-DNJ, D-fagomine (FAG), 2-*O*-α-D-galactopyranosyl deoxynojirimycin (GAL-DNJ), *N*-Methyl-1-Deoxynojirimycin (N-Me-DNJ), 1,4-dideoxy-1,4-imino-(2-*O*-beta-D-glucopyranosyl)-D-arabinitol, 1,4-dideoxy-1,4-imino-D-arabinitol (DAB), and nortropanoline, was reported for the first time [37]. In later studies, more alkaloids, such as aurantiamide acetate, Isofagomine, and 4-*O*-β-D-glucopyranosyl-fagomine (Glu-FAG), were isolated and identified from ML [16,38]. 1-DNJ, FAG, and GAL-DNJ are the major alkaloids, accounting for over 80% of the total alkaloid content in ML. Interestingly, the MLA content varies seasonally. The maximum content of 1-DNJ occurs in summer, the highest FAG level is found in spring, and GAL-DNJ and Glu-FAG contents reach their peak in autumn [39]. Therefore, the collection of medicinal ML ought to be carried out during the appropriate season.

1-DNJ, a type of iminosugar, is the most representative and characteristic MLA and has been extensively investigated. Iminosugars are sugar analogs in which the oxygen atom in the pyranose ring is replaced by a nitrogen atom [40]. Structurally similar to monosaccharides, iminosugars exhibit a unique mechanism of action by modulating the glucose metabolism through the competitive inhibition of various enzymes essential for carbohydrate digestion [4]. To date, numerous studies have confirmed the hypoglycemic effects of 1-DNJ, and its safety and non-toxicity have been validated in clinical trials [13]. For instance, a study based on next-generation sequencing and intestinal microbiota analysis revealed that 1-DNJ decreased the blood glucose level and improved insulin sensitivity in pre-2-diabetic mice, as well as significantly reducing the relative risk of T2DM in prediabetic mice by approximately 83.7% [41]. In vivo and in vitro experiments have indicated that the main anti-diabetic mechanisms of 1-DNJ include the inhibition of carbohydrate-digesting enzymes, improvement in insulin resistance, protection of pancreatic β-cells, regulation of lipid metabolism, alleviation of oxidative stress, and regulation of gut microbiota [10]. 1-DNJ can be extracted from plants, insects, and microbial strains, and it can be synthesized using chemical methods [42]. Among these methods, plant extraction using ML as the raw material is currently the most widely applied and technologically mature approach for 1-DNJ production [40]. Analogous to 1-DNJ, FAG, GAL-DNJ, N-Me-DNJ, and DAB are natural iminosugars that are believed to be intensely effective α-glycosidase inhibitors and may help to reduce the blood glucose levels [43].

#### 2.2.2. Mulberry Leaf Flavonoids (MLF)

Flavonoids are a class of compounds with a unique C6-C3-C6 skeleton structure, consisting of two aromatic rings connected by a three-carbon bridge [4]. It is widely known that flavonoids are the main constituents responsible for the anti-diabetic properties of ML, where they are abundant, accounting for up to 1–3% of the dried ML [44]. The hypoglycemic activity of MLF is primarily attributed to the inhibition of α-glucosidase [19]. Owing to the significant blood glucose-lowering effects of MLF, scholars worldwide are increasingly focusing on their isolation, identification, and mechanistic studies. To date, a series of flavonoids with potent hypoglycemic potential have been isolated and identified from ML, containing rutin, quercetin, isoquercitrin, kaempferol, and astragalin [45]. A recent study revealed that a novel geranylated flavonoid compound from ML, along with its structural analogs, can inhibit the PTP1B protein and enhance glucose uptake in insulin-resistant cells [25].

#### 2.2.3. Mulberry Leaf Polysaccharides (MLP)

Polysaccharides are polymer carbohydrate molecules composed of long chains of monosaccharide units linked by glycosidic bonds, which provide constituent monosaccharides or oligosaccharides during hydrolysis [46]. The monosaccharide composition and structural features of MLP are very complex, but the majority of them are acidic heteropolysaccharides with α, β-glycosidic linkages. They are primarily located within epidermal cells and have molecular weights ranging from 10^3^ to 10^6^ Da [47]. Although the monosaccharide composition of MLP varies across studies, they predominantly consist of galactose (Gal), galacturonic acid (GalA), arabinose (Ara), rhamnose (Rha), glucose (Glc), glucuronic acid (GlcA), mannose (Man), and xylose (Xyl) [48,49]. In addition, monosaccharides such as fucose (Fuc), ribose (Rib), and sorbose (Sor) have also been found in some MLP [33]. The mulberry variety, geographical origins, drying methods, extraction methods, and purification processes may have effects on the average molecular weight, composition, and molar ratio of monosaccharides in MLP, which are related to their bioactivity [50,51]. Consequently, research on the extraction methods and quality control of MLP has obtained increasing attention from scholars worldwide. Published studies demonstrate that MLP are key bioactive constituents in ML, exhibiting notable anti-diabetic effects and significant advantages, such as mild adverse drug reactions [52]. MLP exert anti-diabetic effects through multiple mechanisms and pathways. Among these, numerous studies focus on the role of MLP in regulating gut microbiota to alleviate diabetes [34,53].

#### 2.2.4. Mulberry Leaf Phenolic Acids (MLPA)

Phenolic acids represent one type of the relatively abundant components found in ML. At present, approximately 20 phenolic acids have been isolated from ML, including neochlorogenic acid, cryptochlorogenic acid, chlorogenic acid, caffeic acid, and gallic acid [4,54]. MLPA with chlorogenic acid as the principal constituent can prevent T2DM by inhibiting disaccharidases (sucrase, maltase) and glucose transport [55]. Neochlorogenic acid, an isomer of chlorogenic acid, was not found to directly reduce blood glucose levels. However, neochlorogenic acid has been proved to have an effect on ameliorating diabetic nephropathy based on a db/db mice model, because it effectively inhibits the accumulation of glycation product [56]. Therefore, based on existing preclinical studies, the hypoglycemic activity of MLPA is primarily attributed to chlorogenic acid and its isomers [57].

#### 2.2.5. Mulberry Leaf Proteins (MLPR) and Peptides (MLPP)

The total protein content accounts for 14.40% of the dry weight of ML, mainly including albumin, globulin, gliadin, glutelin, and insoluble protein, which is regarded as an important source of plant protein [44]. Although macromolecular MLPR does not exhibit significant hypoglycemic activity, some of its hydrolysis products (bioactive peptides) can alleviate T2DM by regulating glucose and lipid metabolism and suppressing oxidative stress [35]. The bioactivity of MLPP is influenced by the fermentation methods and the specific proteases used during the hydrolysis process. For example, the neutral proteolytic hydrolysates of MLPR exhibit superior α-glucosidase inhibitory activity compared to hydrolysates produced using complex protease, flavor protease, alkaline protease, trypsin, and papain [1].

#### 2.2.6. The Synergistic Effects of the Constituents

Growing evidence suggests that bioactive components in ML, particularly flavonoids and alkaloids, possess synergistic effects in lowering blood glucose [58,59]. For instance, Ji et al. discovered that the combination of MLA and MLF significantly alleviated diabetic nephropathy in SD rats by modulating the Wnt/β-catenin and TGF-β/Smads signaling pathways. The therapeutic effects of this combination were significantly superior to the use of MLA or MLF only, but their synergistic mechanisms remain unclear [60]. Vitexin, a known flavonoid in ML, has also been proved to possess a synergistic effect in inhibiting α-glucosidase activity when combined with 1-DNJ [20]. In another interesting study, the combination of 1-DNJ and 5,6,7-trihydroxyflavone aglycone was shown to synergistically inhibit α-glucosidase and its MGAM subunit in vitro. Further mechanistic investigations have revealed that the flavonoid aglycone acts as a positive allosteric inhibitor, binding to a non-competitive site on MGAM. This binding induces a conformational change in the MGAM-N active site, enhances the enzyme’s affinity for 1-DNJ, and consequently potentiates the inhibitory effect of 1-DNJ against α-glucosidase [61]. Furthermore, MLF and MLP also demonstrate notable synergy, with their mixture exhibiting superior inhibitory effects on both α-glucosidase and α-amylase compared to the individual components [58]. These studies indicate that the hypoglycemic activity of ML results from the synergistic interactions of various bioactive constituents, including flavonoids, polysaccharides, and alkaloids, which involve multiple targets and pathways [20]. However, the detailed synergistic mechanisms require further investigation.

### 2.3. Extraction Methods Applied in the Bioactive Constituents of ML

**Figure 3:**
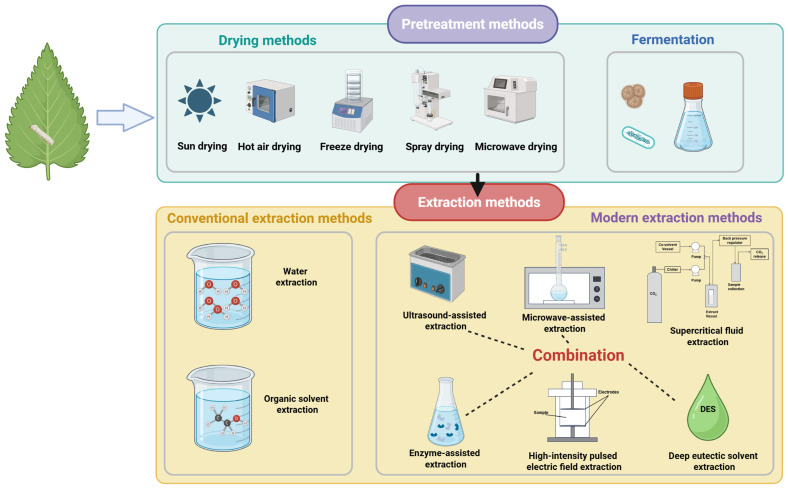
The conventional and modern extraction technologies used to extract hypoglycemic components from ML (created in https://BioRender.com).

ML has abundant bioactive compounds, such as alkaloids, flavonoids, polysaccharides, phenolic acids, and peptides. The appropriate selection of extraction methods is crucial for efficiently extracting and separating hypoglycemic compounds from ML. At present, numerous techniques are employed to prepare ML extracts. Researchers not only choose extraction methods based on the type of target compounds but also focus on optimizing methodologies to enhance extraction efficiency, reduce resource consumption, and minimize environmental impact. Furthermore, response surface methodology (RSM) and machine learning techniques such as artificial neural networks (ANNs) have been employed to investigate the optimal extraction parameters [62]. In the following section, we will provide a detailed introduction to a series of conventional and modern extraction technologies. Each extraction technology has distinct advantages and limitations, as well as varying applicability under different conditions. Additionally, we will discuss how factors such as drying methods and fermentation influence extraction products (Figure 3).

#### 2.3.1. Conventional Technologies

Water extraction and organic solvent extraction are two conventional technologies employed for isolating bioactive compounds from ML. Traditional extraction techniques, primarily maceration and reflux extraction, present certain limitations, including high energy consumption, low extraction yield, and prolonged extraction time. Aqueous extraction is a frequently used technique for separating polysaccharides, alkaloids, and proteins from ML. For MLP, hot water extraction is the most commonly used method due to its operational simplicity and cost-effectiveness. The optimization of extraction parameters primarily focuses on factors such as extraction time, temperature, liquid/solid ratio, and number of extraction cycles. However, there are few studies considering the influence of pH. Additionally, relevant research has indicated that extraction temperature has the greatest impact on polysaccharide yield, followed by the liquid/solid ratio [63]. MLA, which are highly water-soluble, can also be effectively extracted using aqueous solutions [64]. In order to improve the extraction yield of MLA, hydrochloric acid is often added to the aqueous solution to maintain an acidic pH, thereby converting the alkaloids into more soluble salt forms [65]. The extraction of MLPR primarily contain the salting-out method and the alkali–acid precipitation method. Among these methods, the alkali–acid precipitation technique separates proteins by exploiting proteins’ denaturation and aggregation, leading to precipitation at isoelectric points or in strongly acidic or alkaline environments. This method is simple and cost-effective, but the obtained proteins are prone to denaturation, often resulting in relatively low recovery rates and purity. The salting-out method extracts proteins by utilizing varying precipitation thresholds of different substances in a salt solution. Nevertheless, its application in MLPR extraction typically yields low amounts [51]. Organic solvent extraction is frequently utilized to isolate flavonoids from ML, with the choice of solvent being the main factor influencing the extraction efficiency. Ethanol is the most widely used organic solvent, whereas methanol and other solvents are used in few studies [66]. Detailed information regarding conventional technologies, including extraction methods, solvents, conditions, and yields, is summarized in Table 2.

#### 2.3.2. Modern Extraction Technologies

Given the advantages in terms of environmental friendliness, low energy consumption, high extraction yield, and short extraction time, modern extraction methods are garnering increasing attention. At present, the modern methods applied in extracting hypoglycemic components from ML mainly include UAE, MAE, EAE, supercritical fluid extraction (SFE), high-intensity pulsed electric field extraction (PFE), deep eutectic solvent extraction (DEE), and combined extraction methods. Table 3 summarizes the modern extraction techniques, conditions, extraction yields, and advantages and disadvantages for ML.

##### UAE

UAE is a technique that utilizes the cavitation effect induced by ultrasonic waves in a liquid medium to efficiently extract bioactive compounds from plant materials. Ultrasound generates microscopic bubbles by compressing and expanding the liquid. when these bubbles collapse, they produce localized high temperatures and pressures, which break down plant cell structures and improve solvent penetration and the release of compounds [79]. Compared to conventional extraction methods, UAE is a safe, efficient and environmentally friendly extraction technique, which has become a popular choice for extracting polysaccharides, flavonoids, and alkaloids from ML [75]. The efficiency of UAE is determined by a combination of factors, including ultrasonic power, temperature, duration, liquid/solid ratio, solvent, and the physicochemical properties of the target compounds. The influence of ultrasonic power on extraction yield occurs as higher power intensities lead to more pronounced cavitation and mechanical forces, accelerating the transfer of bioactive components from the raw material into the solvent. However, excessively high power can cause the degradation of thermosensitive compounds. Temperature influences extraction efficiency in two opposing ways. Increasing the temperature enhances the diffusion rate and the solubility of active compounds. However, a high temperature also diminishes sonochemical effects and reduces the cavitation of bubble collapse. Prolonging the ultrasonic time can improve extraction yield, but an excessive duration may cause excessive cavitation, cell disruption, and elevated temperature. These factors may increase the degradation of the secondary metabolite [82]. Considering the impact of various factors, research focused on optimizing UAE parameters for extracting bioactive compounds from ML is of significant importance. For 1-DNJ, an ultrasonic-vaccum-assisted extraction technique was demonstrated to produce higher yield compared to standard UAE. When the optimal conditions were applied, the extraction yield of 1-DNJ reached 1.10 ± 0.02 mg/g [81]. For phenolic compounds, the dominant factors affecting yield in UAE are liquid/solid ratio, extraction time and solvent. The optimal extraction conditions are using 40% ethanol as the solvent, an ultrasonic duration of 35 min, and a liquid/solid ratio of 400 mL/g. Under these conditions, the yield of phenolic compounds reaches 36.00 ± 2.00 mg/g [75]. Regarding MLP, extraction temperature is identified as the most significant factor influencing yield, followed by the liquid-to-solid ratio and ultrasonic power. The recommended extraction conditions of MLP are a liquid/solid ratio of 16 mL/g, extraction time of 58 min, extraction temperature of 65 °C, and ultrasonic power of 500 W. These settings result in an MLP yield of up to 14.47% [80].

##### MAE

MAE is an extraction technique that utilizes microwave energy to accelerate the mass transfer process between the sample and the solvent. The principle is that microwave radiation can penetrate materials and accelerate molecular vibrations, thereby generating heat. Instead of traditional heating techniques that rely on convection and thermal conduction, microwave heating operates at the molecular level, leading to a more uniform heating process without temperature gradients [89]. In comparison with traditional hot water extraction, MAE does exhibit numerous advantages, including better reproducibility, shorter extraction time, lower solvent use, and protection of heat-sensitive compounds. For ML, MAE is mainly used to extract polysaccharides, reducing the extraction time from several hours to about 10 min [84]. The optimization of extracting MLP by MAE focuses on parameters, including microwave power, liquid/solid ratio, extraction time, and extraction temperature [63]. For instance, K. Thirugnanasambandham et al. investigated the influence of sample weight, microwave power, and extraction time on polysaccharide yield. They found that the optimal conditions for extraction were an ML sample weight of 20 g, a microwave power of 170 W, and an extraction duration of 10 min. Under these parameters, the MLP yield was 9.41% [83].

##### SFE

SFE is a novel and innovative technique utilized to extract bioactive compounds from various materials. It employs supercritical fluids, which possess unique physicochemical properties, including high diffusivity, low viscosity, and low surface tension. These properties grant supercritical fluids superior permeability into matrix pores, making them an ideal choice for extracting active constituents from plants [90]. Supercritical CO_2_ is the most commonly used supercritical fluid, owing to its relatively low critical temperature (31.1 °C) and other advantageous properties. To date, supercritical CO_2_ has predominantly been applied for the extraction of non-polar triterpenoids from ML, such as β-sitosterol. β-Sitosterol, a major phytosterol found in ML, exhibits notable pharmacological effects, including anti-diabetic, antioxidant, anti-inflammatory, antimicrobial, and immunomodulatory properties. K.A. Santos et al. reported that SFE significantly reduced the extraction time of β-sitosterol to 2 h, compared to the 6 h required by conventional Soxhlet extraction. Nevertheless, due to the pressure limitations of their lab (maximum 200 bar), the yield of β-sitosterol obtained via supercritical CO_2_ extraction reached only 69% of that achieved through Soxhlet extraction. Consequently, further optimization of the extraction parameters for supercritical CO_2_ is necessary to enhance the yield of β-sitosterol [71]. However, for polar compounds present in ML, such as phenolic acids and flavonoids, the extraction effect of supercritical CO_2_ is poor, and the yield is much lower than that of the 70% ethanol extraction method. This might be due to the low polarity of CO_2_ [90]. Future investigations could consider incorporating small amounts of cosolvents, such as ethanol or methanol, to adjust the polarity of CO_2_. This adjustment suggests a possible role in improving extraction efficiency and broadening the applicability of SFE for isolating bioactive constituents from ML.

##### EAE

EAE is extensively acknowledged as a safe and environmentally sustainable extraction technique, characterized by advantages such as high efficiency, a short extraction time, simple procedures, and low energy consumption [85]. Commonly employed enzymes in EAE include proteases, cellulases, amylases, and pectinases, as well as various commercial enzyme mixtures [62]. The efficiency of EAE is influenced by various factors, including the enzyme type, the degree of hydrolysis, the pH, the extraction time, temperature, and the enzyme–substrate ratio. Firstly, the selection of enzyme type is essential for optimizing the extraction process. For example, owing to the specificity of peptide bond cleavage, ML treated by different enzymes demonstrates notable differences in extraction yield, molecular weight, and the bioactivity of MLPR. EAE plays a dual function in protein extraction by both disrupting biological barriers to improve the extraction yield of protein and hydrolyzing proteins into peptides with higher bioactive properties [91]. For MLP, the combined application of pectinase, cellulase, and protease has been proved to increase extraction efficiency. Specifically, the functions of pectinase and cellulase are to promote the solubilization of polysaccharides into the extraction solvent by facilitating the degradation of the plant cell wall, while protease can decompose proteins bound to polysaccharides into amino acids and peptides, which contributes to an increase in polysaccharide purity [85]. Secondly, optimizing the pH value is of importance and the optimizing process should consider enzyme type, as each enzyme exhibits a distinct optimal pH range. Additionally, the structural characteristics and physicochemical properties of the target bioactive compound must be taken into account. For instance, the optimal pH for the cellulase-mediated extraction of 1-DNJ from ML is 3.5–4.5. This range is suitable because 1-DNJ is a piperidine alkaloid, which has higher solubility under acidic conditions. Excessively low pH values can inhibit cellulase activity, causing a decrease in extraction efficiency [65]. It is important to highlight that the improvement in 1-DNJ extraction yield achieved through EAE is not only due to the hydrolysis of the cell wall, which facilitates the release of bioactive constituents, but may also be related to the enzymatic transformation of other alkaloids, such as GAL-DNJ, into 1-DNJ [92]. As the degradation product of GAL-DNJ, 1-DNJ shows comparatively greater oral bioavailability and bioactivity than GAL-DNJ [93]. Furthermore, the release of active compounds can be enhanced by suitably prolonging the digestion duration, elevating the extraction temperature, and optimizing the enzyme–substrate ratio.

##### PFE

PEF is a new method for the extraction of bioactive compounds from plant materials. The principle of PEF is mainly based on the influence of electric fields on cell membranes. When cells are exposed to high-intensity electric fields for a short period of time, the cell membranes will form micropores. This technique can destroy the cell structure at comparatively low temperatures to promote the release of intracellular constituents. Consequently, PEF improves extraction efficiency while maintaining the structural integrity of bioactive compounds. Additionally, PEF is recognized as a cost-effective method due to its relatively low energy requirements and has been extensively employed for the extraction of diverse natural products. However, currently, there are few studies on the extraction of hypoglycemic active components from ML using PEF. Chaiyana W et al. first emphasized that PEF can significantly increase the phenolic content and the radical scavenging activity of ML extracts compared to conventional maceration methods. However, the overall extraction yield of PEF was significantly lower than that obtained through traditional maceration, likely due to the shorter extraction time and fewer extraction cycles [86]. Therefore, further research is essential to optimize the parameters of PEF for the efficient extraction of active constituents from ML.

##### DEE

Deep eutectic solvents (DESs) are a category of green solvents formed through interactions between hydrogen bond donors (HBDs) and hydrogen bond acceptors (HBAs). These solvents exhibit enhanced ability to penetrate plant cellular structures via hydrogen bonding, facilitating the rapid dissolution of bioactive compounds. DESs possess several advantageous properties, including environmental compatibility, widespread availability, straightforward synthesis, biodegradability, low toxicity, high extraction efficiency, and cost-effectiveness. These characteristics make them a promising alternative to conventional toxic organic solvents for the extraction of active constituents [87]. At present, DESs are used to extract polysaccharides, phenols, and alkaloids from ML. The optimal extraction parameters for MLP using DES are a choline chloride/malic acid molar ratio of 1:4, a liquid/solid ratio of 40:1 mL/g, a DES water content of 44%, an enzyme concentration of 3%, an ultrasonic time of 40 min, and an ultrasonic power of 350 W. Under these conditions, the yield of MLP reached 10.20 ± 0.05% [94]. Contributed to the strong multi-interaction between DESs and target components, DESs have obvious advantages in the extraction of phenols from ML compared with conventional solvents such as ethanol and methanol [65]. In conventional extraction technologies, various classes of bioactive components typically coexist in the extract, and subsequent separation and purification steps are necessary. DESs address this issue by synthesizing specific DESs using different HBDs and HBAs, enabling the efficient and selective separation of target components. For instance, R. Ma et al. developed a pH-responsive DES aqueous system capable of simultaneously extracting alkaloids and flavonoids from ML, followed by in situ phase separation to isolate MLF and MLA. Upon mixing the hydrophobic DES with water to form a biphasic system, flavonoids were preferentially enriched in the DES phase, while alkaloids concentrated in the aqueous phase, consistent with the “like dissolves like” principle. This study synthesized five new hydrophobic DESs utilizing long-chain fatty acids (valeric acid, nonanoic acid, hexanoic acid, heptanoic acid, and octanoic acid) as HBDs and 2-methyl-2,4-pentanediol (MPD) as the HBA. Among them, the optimal DES was identified as [HexA][MPD] [88]. Compared to traditional solvents, this DES aqueous system exhibits high extraction efficiency, enhances the purity of the extracts, and demonstrates favorable reusability.

#### 2.3.3. Combined Extraction Techniques

In recent years, a variety of combined extraction techniques have been developed. The combination of different extraction methods is beneficial for overcoming the inherent limitations of each individual approach, thereby promoting the extraction yield and selectivity. Nonetheless, these combined extraction techniques face challenges related to cost-effectiveness, energy consumption, and scalability, which may limit their range of applications. Specifically, the combined extraction of ML often involves EAE coupled with UAE and MAE. Enzyme-ultrasound-assisted coupling extraction (EUCE), an innovative technique, has been employed to extract proteins from ML. The study indicates that the protein content obtained via EUCE is significantly high than that achieved through EAE or conventional alkali–acid precipitation methods, reaching a yield of 62.69%. Compared to conventional extraction methods, the protein extracted by EUCE showed enhanced enzymolysis properties and increased solubility due to alterations in its secondary structure and a reduction in average particle size. The optimal conditions for EUCE were determined to be a pH of 7.20, ultrasonic temperature of 35 °C, cellulase dosage of 4.20%, and ultrasonic time of 10 min [95]. In another study, enzyme-microwave-assisted coupling extraction (EMCE) technique was utilized to promote the extraction efficiency of MLP. This method combines the advantages of EAE and MEA, resulting in a shorter extraction time, a higher extraction yield, and lower costs. The optimal parameters have been identified as a liquid/solid ratio of 15 mL/g, an extraction temperature of 76 °C, and an extraction time of 13 min, resulting in a 15.23% extraction yield of MLP [96]. DEE is also frequently combined with UAE and MAE. For instance, P. Zhou et al. developed a simple, eco-friendly, and efficient method for extracting phenolic compounds from ML using deep eutectic solvent-ultrasound-assisted coupling extraction (DUCE) [97]. A summary of the combined extraction techniques is presented in Table 4.

#### 2.3.4. Influence of Pre-Treatment Methods

In addition to extraction methods, the efficiency of extraction and the activity of hypoglycemic components in ML are influenced by pre-treatment processes such as drying and fermentation.

##### Influence of Drying Methods

Fresh ML has a high moisture content, which accelerates the enzymatic degradation of heat-sensitive compounds and hinders the long-term preservation of ML. Drying is an effective pre-treatment method to reduce moisture content, delay spoilage, and improve the storability of ML. Nevertheless, the choice of drying method can significantly influence the bioactive constituents of ML. Traditional drying methods, such as sun-drying, air-drying and hot air-drying are characterized by simple operation and low energy consumption. However, they require the prolonged heat exposure, which may increase the risk of the degradation of active ingredients. In contrast, emerging drying technologies, including freeze-drying (FD), spray-drying (SD), and microwave-drying (MD), have attracted considerable interest due to their advantages in reducing drying time, improving efficiency, and decreasing the degradation of heat-sensitive compounds. The stability of MLF is particularly susceptible to high temperatures and oxygen exposure. Therefore, FD has been regarded as a good choice for drying ML in order to preserve MLF. For instance, C. Zhao et al. systematically compared the effects of five drying methods (SD, FD, MD, air-drying, and oven-drying) on bioactive compounds in ML, suggesting that rutin content of ML extract dried by FD was significantly higher than that obtained using other drying methods [99]. In another study, the total flavonoid content of ML extract dried by FD was also found to be significantly higher than that of the samples dried by HD. This phenomenon may be attributed to the vacuum conditions during DF, which effectively inhibit the oxidation of flavonoid [72]. Furthermore, compared to air-drying and hot air-drying, FD significantly increases the polysaccharide content of ML extract, changes the composition and molar ratios of monosaccharides, and promotes the antioxidant activity of MLP [50]. Drying methods also influence the alkaloid content of ML extract. Given the high concentration of the representative alkaloid 1-DNJ and the short drying time, MD may be a preferable drying method for alkaloid extraction. However, the optimization of microwave power is essential, as insufficient power prolongs drying time, whereas excessive power can degrade 1-DNJ and diminish its extraction yield [100].

##### Influence of Fermentation

Microbial fermentation, an effective biotechnology for extracting active ingredients from natural sources, is extensively utilized in medicinal development and the food industry. This method uses microbial enzymes to hydrolyze raw materials, which can significantly reduce production costs. Both fungi (e.g., saccharomycetes and molds) and bacteria (e.g., lactic acid bacteria and Bacillus) are common microorganisms employed in fermentation processes. Fungal fermentation serves a dual function in the extraction of flavonoids. Firstly, the hydrolytic enzymes secreted during fermentation break the covalent bonds linking the cell wall to insoluble flavonoids, facilitating the release of MLF from the substrate matrix. Secondly, the fungal secondary metabolism may lead to the biosynthesis of novel flavonoid compounds. Accordingly, several studies have explored the use of fungal fermentation to improve both the extraction efficiency and bioactivity of MLF, focusing on the identification of the optimal fungal strain. For example, solid-state fermentation technology has been demonstrated to facilitate the release and transformation of flavonoids in ML, and the optimal fungal combination for co-fermentation has been determined as *Eurotium cristatum* 5, *Aspergillus cristatus* 6, and *Aspergillus cristatus* 9. However, fermentation with some other fungi may result in a decreased total flavonoid content, because these fungi inhibit and reuse transformed flavonoids, or re-convert them into other colored substances [101]. Notably, fungal fermentation not only plays a pivotal role in disrupting the cell wall of ML but also promotes the dissociation of phenolic compounds and the deglycosylation of flavonoids. For instance, although quercetin and kaempferol are present in minimal amounts in unfermented ML, solid-state fermentation with fungi such as *Monascus anka*, *Monascus purpureus*, and *Aspergillus niger* significantly increases the content of these flavonoid aglycones. Concurrently, the levels of flavonoid glycosides, including astragalin, isoquercitrin, and rutin, decrease, and the fermented ML exhibits higher antioxidant capacity, α-glucosidase inhibitory activity, and antimicrobial properties [102,103]. Moreover, given the low extraction yield of 1-DNJ via conventional methods, microbial fermentation has been employed to augment extraction efficiency. Co-fermentation with *L. fermentum* and *S. cerevisiae* has been recognized as the most effective fermentation method for increasing the content of 1-DNJ in ML [67,104]. Based on the premise that proteases secreted during bacterial fermentation degrade proteins, bacterial fermentation can increase the abundance of bioactive peptides in ML. For example, Zaheer et al. used bacterial strains with high protease activity, such as Bacillus amyloliquefaciens LFB112 and Bacillus subtilis H4, obtaining and identifying three novel antioxidant peptides (FRFDP, RFGG, and GPPLAFGGGP) [105].

### 2.4. Quality Analysis Studies of ML Constituents

**Figure 4:**
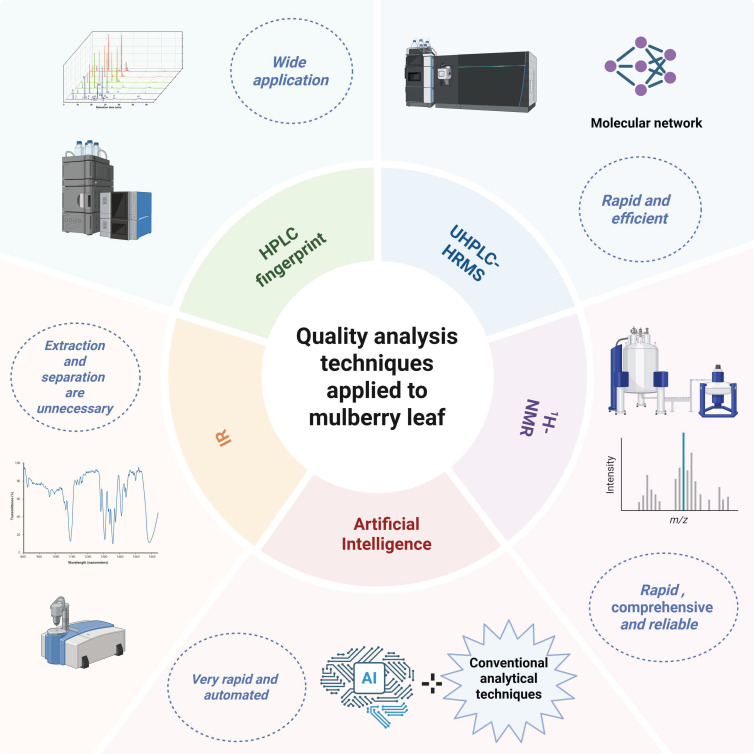
The techniques applied in the quality analysis of ML (created in https://BioRender.com).

The quality of ML is a critical determinant of its hypoglycemic activity and safety. The quality of ML is affected by several factors, including the variety of mulberry tree, the harvest season, the maturity of the leaf, and the techniques employed during processing. Therefore, the establishment of robust quality control standards is crucial for the development and application of ML [66,106,107]. The 2020 edition of the Chinese Pharmacopoeia outlines primary identification techniques for ML, containing macroscopic examination, microscopic identification, TLC, and HPLC. According to the Chinese Pharmacopoeia, the moisture content of ML should not exceed 15%, and the alcohol-soluble extract must be at least 5%. Additionally, the determination of rutin content by HPLC is regarded as a quality marker (Q-Marker). To date, numerous studies have addressed the quality control of ML. Our investigation into quality analysis methods for ML beyond those stipulated in pharmacopeias reveals that these techniques primarily focus on HPLC fingerprint, ultra-high-performance liquid chromatography–high-resolution mass spectrometry (UHPLC-HRMS), infrared spectroscopy (IR), proton nuclear magnetic resonance (^1^H-NMR), and artificial intelligence (Figure 4).

#### 2.4.1. HPLC Fingerprint

Chromatographic techniques employed in the quality assessment of ML predominantly encompass TLC, liquid chromatography (LC), and gas chromatography (GC). As a simple, cost-effective, versatile, and highly specific method, TLC has been used for the authentication of ML according to the Chinese Pharmacopoeia. GC is a well-established method for the analysis of volatile constituents in herbal medicines, and GC-MS has been utilized to construct the fingerprint of ML volatile oil [108]. HPLC is an analytical method characterized by simple operation, broad applicability, and high precision, making it one of the most commonly used techniques in herbal medicine analysis. The HPLC fingerprint can characterize the overall variations in the intrinsic quality of herbs, and Q-Markers are closely associated with their bioactivity. Rutin, a widely recognized Q-Marker for ML, is noted in the current edition of the Chinese Pharmacopoeia and is extensively applied in quality-control practices. The rutin content of qualified ML cannot be lower than 0.1%. However, rutin can be found in numerous plants and herbs, rendering it non-specific as a marker for ML. Additionally, as an individual compound, rutin alone does not adequately reflect the comprehensive quality of ML. Consequently, in order to conduct a more comprehensive evaluation of ML quality, researchers have identified multiple components as Q-Markers and developed detection techniques [109]. For example, X. Ling et al. established an HPLC fingerprint for ML using chlorogenic acid, rutin, and astragalin as Q-Markers, and analyzed the quality of ML samples from different geographic regions and harvest periods [66]. The traditional HPLC fingerprint generally uses a single wavelength for detection. This method is simple to operate but may lead to inaccuracies in identifying chemical constituents. To enhance the analytical accuracy of complex ingredients, multi-wavelength fusion technology has been employed in the quality analysis of ML. For instance, D. Yan et al. developed a multi-wavelength fusion HPLC fingerprint for ML. This method enables the simultaneous quantification of protocatechuic acid, chlorogenic acid, neochlorogenic acid, cryptochlorogenic acid, astragalin, and rutin, providing a more comprehensive assessment of ML quality. They classified the quality grades of ML based on macro-qualitative similarity (Sm), macro-quantitative similarity (Pm), and fingerprint homogeneity (α) under the fused wavelength, with lower ML grades indicating better quality [108]. Moreover, in response to the lack of consensus on analytical techniques and Q-Markers of bioconverted ML, Kim et al. integrated the HPLC fingerprint with bioactivity data to select syringaldehyde, trans-caffeic acid, morin 3-*O*-β-D-glucopyranoside, astragalin, and moracin M 3′-*O*-β-glucopyranoside as Q-Markers. The criteria used to determine the commercial suitability of bioconverted MF for treating diabetes was suggested to be a syringaldehyde concentration of 0.58 µg/g and trans-caffeic acid concentration of 0.46 µg/g [27]. 1-DNJ is one of the most important hypoglycemic compounds in ML, but the lack of ultraviolet absorption makes it difficult to analyze. Although derivatized 1-DNJ can be detected by GC-MS, the previously used trimethylsilyl derivatives have poor thermal stability. To enable the rapid and precise quantification of 1-DNJ in ML, Eruygur et al. developed and validated an HPLC method involving the derivatization of 1-DNJ with 9-fluorenylmethyl chloroformate, followed by quantification using HPLC with fluorescence detection. The limit of detection and limit of quantification of 1-DNJ were 1.07 and 3.27 ng/mL, showing the high sensitivity of this method [110].

#### 2.4.2. UHPLC-HRMS

HPLC and HPLC-MS are commonly employed analytical methods for the quality analysis of ML. However, these techniques are limited by their relatively low resolution and insufficient capacity to provide comprehensive compound information. In contrast, UHPLC-HRMS is a more advanced analytical technique, which possesses high resolution and allows for the precise mass determination of compounds. Common HRMS instruments include Q-Exactive Orbitrap mass spectrometry (Q-Orbitrap-MS) and quadrupole time-of-flight mass spectrometry (Q-TOF-MS). Compared to conventional HPLC-MS, UHPLC-HRM is able to substantially reduce the time required for the qualitative identification of complex mixtures of components in ML through data analysis methods such as mass spectral database-matching and molecular networks [111]. For instance, Sánchez-Salcedo et al. reported that the (poly)phenolic fingerprints of white ML and black ML were constructed using ultra-high-performance liquid chromatography coupled with linear ion trap high-resolution mass spectrometry (UHPLC-LTQ MS), and 31 compounds were identified [112].

#### 2.4.3. IR

FTIR is one of the most frequently used spectroscopic techniques, with each sample producing a distinctive infrared spectrum that serves as a molecular fingerprint. Accordingly, IR has been widely utilized in the identification and quality analysis of herbal medicines, where its quality assessment principle is commonly based on the overall composition rather than specific Q-Markers. For example, Azlah et al. utilized advanced two-dimensional infrared spectroscopy (2D-IR) to identify and classify thirteen varieties of herbal leaves, including ML. In comparison to conventional FTIR, 2D-IR enables the detection of subtle spectral differences and the resolution of overlapping peaks. Their study indicated that the spectral region between 1800 and 900 cm^−1^ is particularly critical for the identification and differentiation of herbal leaves using IR. A notable advantage of IR over alternative quality analysis techniques is that it can analyze both solid and liquid samples without necessitating the complex extraction or separation processes [53]. However, FTIR combined with chemometrics can also be used for a quick screening evaluation of the quality of ML by detecting the 1-DNJ content [113].

#### 2.4.4. ^1^H-NMR

With the development of instrument technology, ^1^H-NMR spectroscopy has been increasingly utilized for the analysis of complex natural samples. Owing to its ability to simultaneously provide both qualitative and quantitative data, ^1^H-NMR is employed to conduct an unbiased assessment of the constituent components within mixtures. In ML, ^1^H-NMR spectroscopy is predominantly applied to the characterization compounds without UV chromophores such as amino acids, organic acids, alkaloids, and carbohydrate. For example, Liang et al. developed a rapid, reliable, and straightforward ^1^H-NMR-based method for the qualitative and quantitative analysis of eleven principal metabolites in aqueous extracts of ML. This approach presents a viable alternative for the quality control and evaluation of ML. In comparison to traditional HPLC-UV, this ^1^H-NMR methodology reliably and non-destructively determines metabolites that lack UV absorption, without the need for any derivatization or separation procedures [114].

#### 2.4.5. Application of Artificial Intelligence in Quality Analysis

In recent years, artificial intelligence has developed rapidly and has been applied across diverse fields. Deep learning techniques have exhibited considerable efficacy in analyzing and extracting valuable information from large data of natural products to solve complex issues. An increasing number of studies emphasize the potential of artificial intelligence combined with traditional analytical methods in herbal medicine quality analysis, and ML is no exception. For instance, Lin et al. developed a comprehensive and bioactivity correlated quality assessment framework for ML based on the HPLC fingerprint and ANN mode. Specifically, their methodology involved generating an HPLC fingerprint, utilizing chemometric techniques and biological assays to identify Q-Markers, and subsequently constructing an ANN model according to the characteristic peaks of Q-Markers [115]. Additionally, another notable investigation demonstrated that near-infrared (NIR) spectroscopy combined with AI algorithms can rapidly evaluate the tenderness of ML. Although this approach is currently applied only in agriculture, it clearly holds significant potential for the quality analysis of medicinal ML [116].

### 2.5. Hypoglycemic Mechanism of ML Constituents

**Figure 5:**
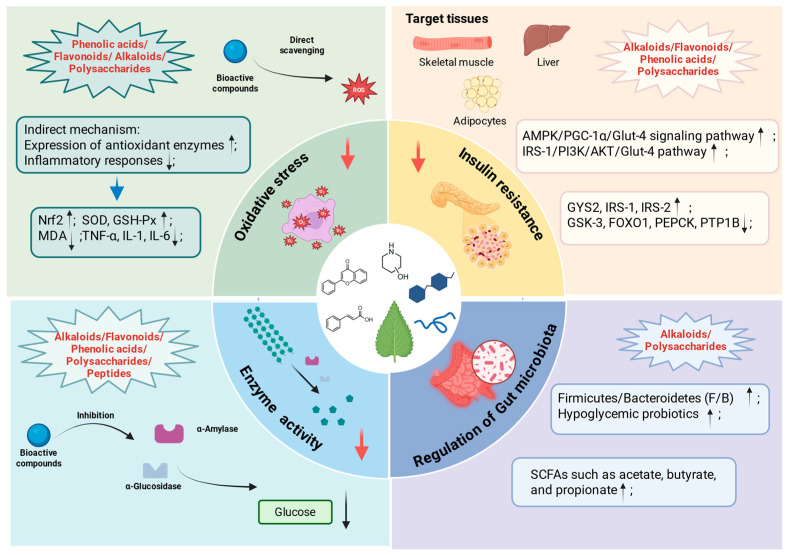
The hypoglycemic mechanisms of ML (created in https://BioRender.com).

ML is a traditional Chinese medicine with a long history of utilization and are effective in reducing blood glucose levels. ML contains a variety of bioactive ingredients that contribute to the regulation of glycemic balance. These constituents operate through multiple mechanisms to sustain glucose homeostasis (Figure 5). The primary mechanisms underlying the hypoglycemic effects of ML are described in detail below and summarized in Table 5.

#### 2.5.1. Inhibition of Oxidative Stress

Oxidative stress is one of the most important factors in the pathogenesis of diabetes and its complications. In diabetes, persistent hyperglycemia and mitochondrial dysfunction facilitates the production and accumulation of reactive oxygen species (ROS). The oxidative stress, mediated by ROS, further aggravates dysregulated blood glucose levels by impairing pancreatic β-cell function and diminishing insulin sensitivity. Recent studies have demonstrated that the abundant natural antioxidants present in ML exert hypoglycemic effects by reducing oxidative stress through both direct and indirect mechanisms. The direct mechanism is defined as the effective scavenging of free radicals, whereas the indirect mechanisms involve modulating antioxidant signaling pathways to increase the expression of antioxidant enzymes, reduce inflammatory responses, and alleviate inflammation-related oxidative damage. Polyphenols are the representative constituents in ML that inhibit oxidative stress. Their phenolic hydroxyl groups endow them with a powerful antioxidant capacity. For example, MLPA such as neochlorogenic acid exhibited a strong free radical scavenging activity and can ameliorate oxidative stress through modulation of the NF-κB signaling pathway [56,126]. Further research indicated that phenolic acids can scavenge ROS in HepG2 under high-sugar culture conditions [117]. 1-DNJ found in ML is not a direct antioxidant, but it can lower postprandial blood glucose by inhibiting α-glucosidase, indirectly reducing high-glucose-induced oxidative stress. Additionally, 1-DNJ can activate Nrf2 in diabetic animals, regulate the production of antioxidant proteins, and prevent inflammation-mediated oxidative stress [10]. Although the antioxidant capacity of MLP alone is relatively modest, they can significantly potentiate the antioxidant effects of flavonoids [79]. Beyond these components, bioactive peptides derived from ML, through fermentation or enzymatic hydrolysis, also exhibit antioxidant properties. Their activities are related to the scavenging of free radical scavenging, their capacity to reduce oxidative stress markers such as MDA and SOD, and their potential to enhance cellular antioxidant defenses via activation of the Keap1-Nrf2 signaling pathway [1,105].

#### 2.5.2. Amelioration of the Insulin Resistance

Insulin resistance represents a common metabolic condition that precedes the development of T2DM, defined as a reduced responsiveness of target tissues (skeletal muscle, liver, and adipocytes) to insulin [8]. Current evidence indicates that extracts derived from ML, along with their bioactive constituents, can enhance insulin secretion, reduce insulin resistance, and improve insulin sensitivity [127,128]. 1-DNJ and flavonoids are the main active ingredients in ML that ameliorate insulin resistance. In animal studies, 1-DNJ significantly alleviated insulin resistance via the activation of the insulin signaling PI3K/AKT pathway in skeletal muscle [120]. In skeletal muscle cells, both 1-DNJ and MLE have been proved to ameliorate insulin resistance effectively by activating the IRS-1/PI3K/AKT signaling pathway. A further mechanistic investigation revealed that 1-DNJ can upregulate p-GSK3β activity through activation of the aforementioned signaling pathway, and p-GSK3β can inhibit p-GS levels to promote glycogen storage in skeletal muscle [121]. Additionally, ML polyphenol-enriched extracts can promote glucose uptake and transport by activating the IRS-1/PI3K/Glut-4 signaling pathway [122]. MLF can also reverse insulin resistance in skeletal muscle by increasing glucose uptake and mitochondrial function through the regulation of the AMPK/PGC-1α/Glut-4 signaling pathway [8]. Given that skeletal muscle is the primary insulin-responsive tissue, responsible for approximately 80% of insulin-mediated glucose uptake, it plays a pivotal role in maintaining systemic glucose homeostasis. Consequently, the majority of research has concentrated on elucidating the effects of ML extracts and their active constituents on skeletal muscle insulin sensitivity. Nonetheless, some studies pay attention to insulin resistance in other target tissues, such as liver and adipose tissue. For instance, Sangtong alkaloids composed of the total MLF and MLA, were able to significantly upregulate the IRS-1/PI3K/AKT/Glut-4 signaling pathway in the hepatic tissue of diabetic mice, reduce insulin resistance, and ameliorate pathological alterations in pancreatic tissue [123]. In a Caco-2/insulin-resistant HepG2 model, ML polyphenols, including luteoforol and p-coumaric acid, alleviated hepatic insulin resistance by modulating the expression of genes associated with insulin signaling (IRS1 and AKT), glycogen synthesis (GYS2 and GSK-3β), and gluconeogenesis (FOXO1 and PEPCK) [5]. Furthermore, MLP have emerged as potential agents for ameliorating insulin resistance. A study demonstrated that MLP can ameliorate hepatic insulin resistance by regulating the expression of two antagonistic regulators within the insulin signaling pathway: protein tyrosine phosphatase 1B (PTP1B) and insulin receptor substrate 2 (IRS-2). Specifically, the downregulation of PTP1B coupled with the upregulation of IRS-2 contributed to the positive modulation of insulin signaling in liver tissue [2].

#### 2.5.3. Regulation of Related Enzymes Activity

α-Glucosidase and α-amylase are enzymes involved in carbohydrate digestion that can rapidly increase postprandial blood glucose levels. Inhibiting the activities of these carbohydrate-digesting enzymes and delaying the release of glucose from carbohydrates constitute an important hypoglycemic mechanism of ML, which contains various active components, such as alkaloids, flavonoids, polysaccharides, and bioactive peptides [129]. 1-DNJ is the most important MLA, with the inhibition activities of α-glucosidase and α-amylase. It is well known that 1-DNJ has a similar chemical structure to glucose and binds more readily to glucosidases than glucose itself. Its mechanism of inhibiting intestinal α-glucosidase activity is considered to involve competition with glucose for the enzyme’s active site [130]. 1-DNJ is a potent α-glucosidase inhibitor, with an enzyme-inhibitory activity comparable to clinically used α-glucosidase inhibitors such as acarbose, voglibose, and miglitol [9]. Other MLA, such as FAG and GAL-DNJ, also exhibit inhibitory activity against carbohydrate-digesting enzymes, but their effects are significantly weaker than those of 1-DNJ [14]. Flavonoids are also major components in ML, responsible for inhibiting α-glucosidase and α-amylase. For MLF, astragalin has attracted researchers’ attention as an effective α-glucosidase inhibitor, with the lowest IC_50_ (154.5 μM) among the main polyphenols in ML. Its inhibition mechanism has been shown to involve binding to α-glucosidase at a single inhibitory site, which alters the enzyme’s microenvironment and conformation, reduces the α-helix content, and consequently decreases enzyme activity [19]. Furthermore, several prenylated flavonoids isolated from ML have demonstrated great α-glucosidase inhibitory activity in vitro. Among them, Sanggenon W exhibited an even lower IC_50_ than that of acarbose and showed significant postprandial blood glucose-lowering ability in vivo, highlighting its research value [23]. Bioactive peptides obtained through the enzymatic hydrolysis of MLPR may also serve as potential α-glucosidase inhibitors, with an inhibitory activity closely related to the type of hydrolases, the molecular weight of the peptide, and the amino acid sequence of the peptide [35]. Notably, hydrolysates produced by neutral protease showed the strongest inhibition against α-glucosidase, likely due to their high content of hydrophobic and aromatic amino acids such as leucine, proline, and alanine [1]. Depending on the extraction and purification methods used, MLP exhibit significant variations in monosaccharide composition and biological activity. Some MLP demonstrate inhibitory effects on α-glucosidase and α-amylase activities, while others do not [131].

#### 2.5.4. Regulation of Gut Microbiota

An increasing amount of evidence suggests that the gut microbiota plays a crucial role in regulating the development of diabetes. When the gut microbiota is in a state of dysbiosis, abnormal metabolites and immunomodulatory functions may contribute to metabolic syndrome-like diseases such as T2DM [132]. Current research indicates that modulating the gut microbiota is the main hypoglycemic mechanism of ML and its active components. Animal studies have demonstrated that ML extract ameliorates gut microbiota composition and glucose homeostasis in T2DM mice, primarily through normalization of the quantity, abundance, and distribution of gut microbiota [124,133]. Among the gut microbiota-modulating components in ML, 1-DNJ is the most representative active substance, mainly functioning in two ways. Firstly, 1-DNJ effectively alleviates diabetes-induced gut microbiota dysbiosis. In prediabetic mice modal, high-fat diet treatment increased the abundance of *Enterobacteriaceae*, *Enterococcaceae* and decreased the abundance of *Bifido-bacterium*, *Bacteroides*, *Lactobacillus*, and *Akkermansia*, which was ameliorated by intervention with 1-DNJ. An increased abundance of *Enterobacteriaceae* and *Enterococcacea* may exacerbate the destruction of the intestinal barrier, while the increased abundance of *Bifidobacterium*, *Bacteroides*, *Lactobacillus*, and *Akkermansia* protected the intestine from pathogenic bacteria, and maintained the epithelial cell tight junction. Secondly, 1-DNJ can increase the levels of short-chain fatty acids (SCFAs) such as acetate, butyrate, and propionate, suggesting a potential role in improving glucose homeostasis, reducing intestinal permeability, and mitigating inflammatory reaction [125]. MLP exhibit similar regulatory effects on diabetic gut microbiota. They enhanced gut microbiota diversity and impacted the Firmicutes to Bacteroidetes (F/B) ratio in an HFD-induced mice model [32]. Furthermore, given the interactive relationship between bile acids and gut microbiota, where they mutually regulate each other’s dynamics, MLP can improve glucose and lipid disorders through the gut microbiota–bile acid metabolism pathway [33]. Oligosaccharides derived from the enzymatic hydrolysis of MLP may also possess gut microbiota-modulating properties. MLO 2-1, an oligosaccharide purified from enzymatically hydrolyzed MLP, has been reported to alleviate T2DM because it could selectively promote the proliferation of the hypoglycemic probiotic bacterium Ligilactobacillus murinu [34].

### 2.6. Limitations and a Future Research Agenda

Based on the integration and results of previous studies, the hypoglycemic effects of ML and its bioactive constituents are strongly supported. However, some limitations still need to be addressed. Firstly, the optimization of extraction parameters mainly use RSM, which may not be suitable for complex systems and multi-objective optimization. Secondly, the evaluation of extraction methods is not comprehensive enough, and few studies focus on the impact of different extraction methods on the structure and biological activity of ML’s ingredients. Thirdly, there is lack of quality-control studies and standards for active macromolecules, including polysaccharides and peptides. Fourthly, clinical studies on the bioactive constituents of ML are still extremely scarce. Finally, current research on the gut microbiota predominantly analyzes at the phylum and genus levels; however, investigations at the strain level remain limited.

These limitations highlight the significance of future research in these emerging fields to address existing gaps. Artificial intelligence algorithms such as ANN and Particle Swarm Optimization (PSO) have the advantage of handling highly nonlinear problems and may be widely applied in the optimization of extraction methods in the future to quickly find theoretically optimal parameters. In order to reach a more comprehensive and reliable conclusion, the evaluation of extraction methods should consider their impact on the structure and activity of the extracted components. Future investigations should prioritize the establishment of chemical fingerprint profiles and quality control standards for priority compounds and standardized extracts to ensure the consistency and reproducibility of the research materials. In addition, some polysaccharides and peptides from ML have been identified as bioactive ingredients and hold potential for use as new quality control markers. Future investigations also need to systematically examine the dose–response relationship during both the preclinical and clinical phases to identify the minimum effective dose, 50% effective dose, and maximal effect. This research will facilitate the provision of accurate recommendations for clinical practice. Employing advanced high-resolution methodologies, such as metagenomics, to perform comprehensive analyses of gut microbiota alterations at the strain level both pre- and post-intervention holds promise for elucidating the precise mechanisms underlying the effects of ML extract and its bioactive constituents.

## 3. Methods

### 3.1. Eligibility Criteria

This review summarized the studies on the constituents of ML for treating diabetes. Articles and review articles published in English and other languages between 2015 and 2025 were considered for inclusion. Studies were eligible for inclusion when a full text was available and targeted the research question. Patents, meetings, case reports, and gray literature were excluded. Studies were categorized according to the specific classes of hypoglycemic compounds, including alkaloids, flavonoids, polysaccharides, phenolic acids, and peptides.

### 3.2. Information Sources and Search Strategy

An advanced literature search was performed utilizing the Web of Science and PubMed databases to find relevant articles on the constituents of ML that are involved in regulating blood glucose levels. This systematic review followed the PRISMA 2020 guidelines. In brief, the search strategy incorporated specific keywords related to ML, diabetes, and glucose regulation. Since this review focused on extraction methods, quality control, and mechanisms of action, a supplementary search was carried out using relevant terms. The final literature search was conducted on 3 August 2025. The full search strategy and terms used are presented in Table 6.

### 3.3. Selection and Data Collection Process

After the literature search in the Web of Science and PubMed databases, all collected data were critically analyzed for eligibility following the PRISMA 2020 guidelines. Firstly, the general information of each study, including the title, abstract, journal name, publication year, and DOI, was exported to a Microsoft Excel spreadsheet, and duplicate entries were removed. Subsequently, the articles were screened according to predefined inclusion and exclusion criteria. The systematic evaluation and screening of the literature were performed by two independent reviewers. In cases of discrepancies between the reviewers, consensus was reached through discussion.

## 4. Conclusions and Perspectives

As a traditional Chinese medicinal herb, ML possesses significant hypoglycemic effects and contains various active components, including alkaloids, flavonoids, polysaccharides, and phenolic acids. The glucose-lowering properties of ML and its bioactive compounds are predominantly supported by preclinical studies. However, the hypoglycemic activity of MLE and 1-DNJ has been substantiated by results from clinical trials. The implementation of standardized extraction and analytical methods is essential for the precise assessment of hyperglycemic potential of ML, as well as for ensuring the rigor, reliability, and reproducibility of clinical investigations. Contemporary research increasingly focuses on green and efficient extraction technologies that align with global sustainability objectives. To date, several advanced extraction techniques have been developed and applied to ML, such as EAE, UAE, MAE, DEE, PFE, SFE, and combined extraction methods. Each of these techniques exhibits unique advantages and limitations, and appropriate methods can be selected based on the properties of different components and the purpose of extraction. Additionally, ML extraction is influenced by pretreatment methods, such as drying and fermentation. Methods such as FD, MD, and SD significantly reduce the time required to obtain stable, storable dried ML. Fermentation is an ideal choice for generating novel bioactive peptides from ML. In terms of quality analysis, UHPLC-HRMS demonstrates exceptional sensitivity, making it suitable for the rapid analysis of numerous compounds in ML. Furthermore, some studies combine conventional chromatographic and spectroscopic analytical methods with artificial intelligence, revealing new research directions in ML quality control.

However, the intricate composition of bioactive constituents in ML presents significant challenges for their extraction methods, quality control, and action mechanism investigations. To address these challenges, future investigations should utilize advanced methodologies, including multi-omics analyses and metagenomic techniques, to comprehensively and systematically explore the underlying mechanisms of the diverse compounds present in ML. Moreover, artificial intelligence technology has high application value in optimizing extraction parameters and establishing quality control standards. Furthermore, the majority of existing research on ML is based on cellular and animal models, more clinical validation is necessary in the future.

## Figures and Tables

**Table 1:** Classes and names of hypoglycemic compounds identified from ML.

Class	No.	Compounds	Hypoglycemic Mechanisms	Types of Experiments	Ref.
Alkaloids	1	1-DNJ	Inhibits the activities of α-glucosidase, α-amylase, maltase, sucrase, and iso-maltase; decreases the intestinal absorption of glucose; suppresses insulin resistance; preserves islet β-cells and releases insulin from β-cells; Regulates lipid metabolism; anti-inflammatory and antioxidant properties; regulates gut microbiota; increases PPARγ, C/EBPα, and SREBP-l expression in 3T3-L1 cells; regulates AGEs/RAGE and p38 MAPK/NF-κB pathways	In vitro, in vivo, and clinical experiments	[10,11,12,13,14]
	2	FAG	Inhibits the activities of α-glucosidase; potentiates insulin secretion; suppresses insulin resistance; prevents basal and glucagon-stimulated glycogen degradation;	In vitro and in vivo experiments	[14,15]
	3	GAL-DNJ	Inhibits the activities of maltase, sucrase, and iso-maltase	In vitro experiment	[14]
	4	*N*-Me-DNJ	Inhibits the activity of α-glucosidase	In vitro experiment	[15]
	5	DAB	Inhibits the activities of α-glucosidase and isomaltase	In vitro experiment	[15]
	6	Isofagomine	Inhibits the activity of α-glucosidase	In vitro experiment	[16]
	7	4-*O*-β-D-Glc-fagomine	Inhibits the activity of α-glucosidase	In vitro experiment	[16]
Flavonoids	1	Rutin	Inhibits the activity of α-glucosidase; inhibits the expression of COX-2 and 5-LOX; regulates arachidonic acid (ARA) metabolism disorder	In vitro and in vivo experiments	[17,18]
	2	Isoquercitrin	Inhibits the activity of α-glucosidase; inhibits the expressions of COX-2 and 5-LOX, regulates arachidonic acid (ARA) metabolism disorder; increases PPARγ, C/EBPα, and SREBP-l expression in 3T3-L1 cells; regulates AGEs/RAGE and p38 MAPK/NF-κB pathways	In vitro experiment	[12,17,18]
	3	Kaempferol-3-*O*-rutinoside	Inhibits the activity of α-glucosidase	In vitro experiment	[17]
	4	Astragaloside	Inhibits the activity of α-glucosidase;	In vitro experiment	[17]
	5	Epicatechin	Inhibits the activity of α-glucosidase	In vitro experiment	[19]
	6	Catechin	Inhibits the activity of α-glucosidase	In vitro experiment	[19]
	7	Vitexin	Inhibits the activity of α-glucosidase	In vitro experiment	[20]
	8	Quercetin-3-*O*-glucuronide	Inhibits the activity of α-glucosidase	In vitro experiment	[21]
	9	Quercetin	Inhibits the activities of α-glucosidase and α-amylase	In vitro experiment	[21]
	10	Quercetin 3-(6-malonylglucoside)	Inhibits the activity of α-glucosidase	In vitro experiment	[22]
	11	Kaempferol	Inhibits the activity of α-glucosidase	In vitro experiment	[5]
	12	Mortatarin F	Inhibits the activity of α-glucosidase	In vitro experiment	[23]
	13	Sanggenon W	Inhibits the activity of α-glucosidase	In vitro experiment	[23]
	14	Mortatarin G	Inhibits the activity of α-glucosidase	In vitro experiment	[23]
	15	Morusinol	Inhibits the activity of α-glucosidase	In vitro experiment	[23]
	16	Morusin	Inhibits the activity of α-glucosidase; facilitates glucose consumption and represses the gene expression of ADORA1 and PPARG in L02 cells	In vitro and in vivo experiments	[23,24]
	17	Kuwanon C	Inhibits the activity of α-glucosidase; represses the gene expression of ADORA1 and PPARG	In vitro and in vivo experiments	[23,24]
	18	5,7,2′,4′-tetrahydroxy-3-geranylflavone	Inhibits the activity of α-glucosidase	In vitro experiment	[23]
	19	3′-geranyl-3-prenyl-2′,4′,5,7-tetrahydroxyflavone	Inhibits the activity of α-glucosidase; inhibits tyrosine phosphatase 1B and ameliorate insulin resistance	In vitro experiments	[23,25]
	20	5′-geranyl 5,7,2′,4′-tetrahy-droxyl-flavone	Inhibits the activity of α-glucosidase	In vitro experiment	[23]
	21	Sanggenone K	Inhibits the activity of α-glucosidase	In vitro experiment	[23]
	22	Cyanidin-3-glucoside	Increases glucose consumption	In vitro experiment	[6]
	23	Cyanidin-3-rutinoside	Inhibits the activity of α-glucosidase	In vitro experiment	[6]
	24	Morin	Improves glycogen synthesis, inhibits gluconeogenesis, and augments the Akt and insulin receptors’ phosphorylation; induces oxidative stress	In vitro and in vivo experiments	[26]
	25	Astragalin	Inhibits the activity of α-glucosidase	In vitro experiment	[25]
	26	3′-(2*E*)-7-hydroxy-3,7-dimethyl-2-octen-1-yl-3-prenyl-2′,4′,5,7-tetrahydroxyflavone	Inhibits tyrosine phosphatase 1B and ameliorates insulin resistance	In vitro experiment	[25]
	27	Luteoforol	Ameliorates insulin resistance; inhibits glucose uptake and transport	In vitro experiments	[5]
	28	Luteolin	Reduces glucose uptake by inhibiting GLUT2	In vitro experiments	[5]
Phenolic acids	1	Caffeic acid	Promotes insulin release from β cells; suppresses insulin resistance; regulates insulin signal pathway	In vitro experiments	[27]
	2	Chlorogenic acid	Inhibits the activities of α-amylase and α-glucosidase; lowers oxidative stress	In vitro experiments	[28,29]
	3	Neochlorogenic acid	Inhibits the expressions of COX-2 and 5-LOX; regulate arachidonic acid (ARA) metabolism disorder; inhibits the activity of α-glucosidase	In vitro experiments	[5,18]
	4	Mulberroside A	Inhibits the activity of α-glucosidase; lowers oxidative stress	In vitro experiments	[28]
	5	Syringaldehyde	Inhibits the activity of α-amylase; increases glucose utilization and insulin sensitivity	In vitro experiments	[27]
	6	7-hydroxycoumarin	Inhibits the activity of α-glucosidase; lowers oxidative stress	In vitro experiments	[28]
	7	Benzoic acid	Inhibits the activity of α-glucosidase	In vitro experiment	[19]
	8	Vanillic acid	Inhibits the activity of α-glucosidase	In vitro experiment	[19]
	9	P-coumaric acid	Induces oxidative stress and protects pancreatic β cells, ameliorates insulin resistance; inhibits glucose uptake and transport	In vitro experiments	[3,5]
	10	Gallic acid	Inhibits the activities of α-amylase and α-glucosidase; increases glucose uptake and enhance	In vitro experiments	[29]
	11	Cryptochlorogenic acid	Inhibits the activity of α-glucosidase	In vitro experiment	[30]
Polysaccharides	1	MP4	Inhibits the activity of α-glucosidase; regulates gut microbiota disorder	In vitro experiment	[31]
	2	MP	Improves metabolic disturbance; suppresses insulin resistance; regulates gut microbiota composition	In vivo experiments	[32]
	3	MLP	Ameliorates glucose and lipid metabolism disorders via the gut microbiota–bile acids metabolic pathway	In vivo experiments	[33]
	4	MP	Regulates glucose, amino acid, and lipid metabolism	In vitro and in vivo experiments	[12]
	5	MLO 2-2	Selectively promotes the growth of gut microbiota	In vitro and in vivo experiments	[34]
	6	MLPII	Ameliorates hepatic glucose metabolism and insulin signaling	In vivo experiment	[2]
Pepetides	1	MLPH	Regulates glycolipid metabolism; ameliorate oxidative stress	In vitro and in vivo experiments	[1]
	2	AAGRLPGY	Inhibits the activity of α-glucosidase	In vitro experiment	[35]
	3	VVRDFHNA	Inhibits the activity of α-glucosidase	In vitro experiment	[35]
	4	RWPFFAFM	Inhibits the activity of α-glucosidase	In vitro experiment	[35]
Others	1	(−)-syringaresinol-4-*O*-glucoside	Inhibits the expressions of COX-2 and 5-LOX; regulates arachidonic acid (ARA) metabolism disorder	In vitro experiment	[18]
	2	Chalcomoracin	Inhibits the activity of α-glucosidase	In vitro experiment	[36]
	3	Resveratrol	Regulates glycolipid metabolism; suppresses insulin resistance	In vitro experiment	[29]
	4	Oxyresveratrol	Inhibits the activity of α-glucosidase; reduces oxidative stress	In vitro experiment	[19]

**Table 2:** Conventional technologies used to extract hypoglycemic components from ML *.

Components	Extraction Method	Extraction Conditions					Yield/Products	Ref.
		Solvent	Temperature (°C)	Liquid/Solid Ratio (mL/g)	Extraction Time (min)	Other Conditions		
Alkaloids	Vortex extraction	0.05M HCl	ND	15:1	30	2 times cycle	1-DNJ was 3.38 mg/g	[67]
Alkaloids	Maceration extraction	H_2_O	80	ND	120	2 times cycle	1-DNJ was 1.91 mg/g	[67]
Alkaloids	Reflux extraction	70% ethanol	ND	ND	120	2 times cycle	1-DNJ was 3.77 mg/g	[67]
(Poly)phenols	Maceration extraction	70% ethanol	60	100	60	ND	TPC was 60.10 mg GAE/g DW	[68]
(Poly)phenols	Maceration extraction	71.75% ethanol	67.1	23.2	150	ND	Flavonoids yield was 2.37%	[22]
(Poly)phenols	Maceration extraction	90% ethanol	Roon temperature	ND	4320	ND	TPC was 67.66 mg GAE equivalent/g dry extract, TFC was 39.24 mg rutin equivalent/g dry extract	[69]
(Poly)phenols	Maceration extraction	75% methanol	80	10	120	ND	Content of rutin was 0.32 mg/mL	[8]
(Poly)phenols	Maceration extraction	100% methanol	60	100	60	ND	TPC was 45.50 mg GAE/g DW	[68]
(Poly)phenols	Maceration extraction	80% acetone	ND	25	10	Two cycles	Free phenolic compounds	[70]
(Poly)phenols	Maceration extraction	Ethyl acetate	Room temperature	12	90	Hydrolysis in 2M NaOH (10 mL/g) before extraction	Bound phenolic compounds	[70]
Polysaccharides	Hot water extraction	H_2_O	80	40	60	ND	11.30%	[46]
Polysaccharides	Hot water extraction	H_2_O	92	34	210	ND	10.00 ± 0.50%	[48]
Polysaccharides	Hot water extraction	H_2_O	100	15	180	ND	7.20%	[46]
Phytosterol	Soxhlet extraction	n-hexane	68	ND	6 (h)	ND	β-sitosterol was 2.27 mg/g	[71]
MLA and MLF	ND	60% ethanol	60	30	60	pH 6, two cycles	1-DNJ was 0.14 mg/g, TFC was 19.32 mg rutin equivalent/g DW	[72]
Combined extraction of MLP, MLF and MLA	Maceration extraction	Step 1: H_2_O Step 2: Ethanol–HCl	Step 1: 81 Step 2: ND	Step 1: 20 Step 2: 25	Step 1: 93 Step 2: 142	Step 1: four cycles; Step 2: three cycles	Step 1: MLP yield was 15.57%, MLF yield was 2.69%; Step 2: MLA yield was 0.40%	[73]

* TPC: Total polyphenol content; TFC: total flavonoid content; mg GAE/g DW: milligram of gallic acid equivalent per gram of dry weight; ND: No data.

**Table 3:** Modern extraction technologies used to extract hypoglycemic components from ML *.

Extraction Technique	Components	Extraction Conditions					Yield/Products	Advantages and Limitations	Ref.
		Solvent	Temperature (°C)	Liquid/Solid Ratio (mL/g)	Extraction Time (min)	Other Conditions			
UAE	(Poly)phenols	80% methanol (1% formic acid)	ND	5	25	Three cycles	TPC was 16.13 mg GAE/g DW	Short extraction time, efficient, friendly to heat-sensitive compounds, wide applicability	[74]
	(Poly)phenols	59% methanol	ND	48	77	Ultrasonic power 240 W	The extraction yields of chlorogenic acid, rutin and astragalin were 0.33%, 0.57%, and 0.89%, respectively		[66]
	(Poly)phenols	40% ethanol	ND	400	35	Ultrasonic frequency 35 kHz	Sum of phenolic compounds was 37.30 ± 0.70 mg/g DW		[75]
	(Poly)phenols	70% ethanol	ND	100:3	60	Ultrasonic power 400 W	TPC was 8.33 mg GAE/g DW		[76]
	(Poly)phenols	Ethanol–HCl–water (7:2:1)	75	40	60	Ultrasonic frequency 40 kHz	Quercetin and kaempferol were 6.91 mg/mL and 2.06 mg/mL, respectively.		[77]
	(Poly)phenols	H_2_O	55	85	5	Ultrasonic power 49 W/cm^2^	TPC was 21.78 ± 0.50 mg GAE/g DW, TFC was 11.70 ± 0.26 mg Catechin/g DW		[78]
	Polysaccharides	H_2_O	57	53	80	Ultrasonic power 100 W	6.92 ± 0.29%		[79]
	Polysaccharides	H_2_O	65	16	58	Ultrasonic power 500 W	14.47%		[80]
	Alkaloids	69% ethanol	65	40	25	Ultrasonic power 480 W	1-DNJ was 1.10 ± 0.02 mg/g		[81]
	Alkaloids	H_2_O	29	20	5	Ultrasonic power 60 W, pH 5.98	1-DNJ was 4.10 mg/g		[82]
	Protein	5 g/L NaOH	40	ND	10	Ultrasonic wave 40 HZ	ND		[35]
MAE	Polysaccharides	H_2_O	ND	ND	10	Microwave power 170 W	9.41%	Short extraction time, low solvent consumption, expensive equipment	[83]
	Polysaccharides	H_2_O	60	25	11	Microwave power 263 W	9.50%		[84]
SFE	Phytosterol	supercritical CO_2_	60	ND	120	Pressure 200 bar	β-sitosterol yield was 1.56 mg/g	Short extraction time, eco-friendly, low solvent consumption, efficient in extraction of nonpolar compounds, expensive equipment	[71]
EAE	(Poly)phenols	80% methanol	ND	10	ND	Enzyme: zympex-014, enzyme content 5%, enzymolysis pH 8.5, enzymolysis temperature 70 °C, enzymolysis time 40 min	The yield of crude extract was 3.00 mg/g DW	Efficient, friendly to heat-sensitive compounds, eco-friendly, high selectivity, high cost, long reaction time	[62]
	Alkaloids	Cellulase solution (3.40 mg/mL)	60	1000:17	60	pH 3.8	Extraction yield of 1-DNJ was 0.10%		[65]
	Polysaccharides	H_2_O	85	30	60	Enzyme: pectinase and protease, enzymolysis temperature 45 °C, enzymolysis time 50 min	24.04 ± 0.98%		[85]
PFE	(Poly)phenols	95% ethanol	ND	5	20	PEF frequency 5 Hz, pulse width 1 us	TPC was 71.50 ± 0.90 mg GAE equivalent/g dry extract	Short extraction time, low solvent consumption, efficient, high bioactivity, eco-friendly, expensive equipment, limited applicability	[86]
DEE	(Poly)phenols	Choline chloride/glycerol (1:2 molar ratio)	66	20	35	20% water content	The extraction yield of (Poly)phenols was lower than DES-MAE	Eco-friendly, sustainable, high selectivity, difficult operation	[87]
	Flavonoids and alkaloids	[HexA][MPD] (1:1 molar ratio)	ND	10	80	50% water content, pH 6.50	A product achieved in situ separation of flavonoids and alkaloids		[88]

* TPC: Total polyphenol content; TFC: total flavonoid content; mg GAE/g DW: milligram of gallic acid equivalent per gram of dry weight; ND: No data.

**Table 4:** Combined extraction technologies used to extract hypoglycemic components from ML.

Extraction Technique	Components	Extraction Conditions					Yield/Products	Ref.
		Solvent	Temperature (°C)	Liquid/Solid Ratio (mL/g)	Extraction Time (min)	Other Conditions		
DES-MAE	(Poly)phenols	Choline chloride/glycerol (1:2 molar ratio)	66	20	18	Microwave power 660 W, 20% water content	The extraction yield of (poly)phenols was higher than DEE	[87]
	(Poly)phenols	Choline chloride/citric acid (2:1 molar ratio)	40	50	30	25% water content	The extraction yield of (poly)phenols was 22.66 mg/g	[97]
DES-UAE	(Poly)phenols	Choline chloride/glycerol (1:2 molar ratio)	66	20	35	Microwave power 250 W, 20% water content	The extraction yield of (poly)phenols was lower than DES-MAE and DEE	[87]
DES-EAE-UAE	Polysaccharides	Choline chloride/malic acid (1:4 molar ratio)	ND	40	40	Microwave power 350 W, 44% water content, 3% complex enzyme	10.20 ± 0.05%	[94]
USBE	(Poly)phenols	Na_2_HPO_4_–citric acid buffer solution	49	30	97	Sequentially add buffer solutions with pH values of 2.2, 7.6, and 8.4 as the extraction solution, ultrasonic power 400 W	TFC was 38.23 rutin equivalent mg/g DW	[98]
EUCE	Peptides	NaOH 0.125 M	40	37	40	Ultrasonic power 480 W, neutral protease treated at temperature 45 °C for 2 h after UAE	Enzymatic hydrolysis elevated total amino acid content from 558.53 mg/g to 622.42 mg/g	[1]
	Protein	H_2_O	35	ND	10	pH 7.2, cellulase, enzyme dosage 4.20%	Extraction yield of MLPR was 13.87 mg/mL	[95]
EMCE	Polysaccharides	H_2_O	76	15	13	Enzyme: cellulase, enzyme content 2%, enzymolysis pH 6, enzymolysis temperature 50 °C, enzymolysis time 20 min	15.23%	[96]

**Table 5:** Mechanistic mapping of ML constituents: models, assays, and effects.

Mechanisms	Extract/Bioactive Constituents	Models	Assays	Effects	Ref.
Inhibition of oxidative stress	Ethanol extract of *M. alba* leaves	STZ-induced adult male Wistar rats	MDA, GSH, CAT, SOD and GPx	In the retina of diabetic rats, is decreases MDA, and increases GSH, GPx, SOD, and CAT	[69]
	Phenolics	HePG2 cell	ROS	Reduces the level of ROS in HepG2 under high-sugar culture conditions	[117]
	Neochlorogenic acid	Male db/db mice	Western blot	Modulates NF-κB signaling pathway by reducing p-NF-κB and p-IκB levels	[56]
	Polysaccharides	STZ-induced SD rats	MDA, SOD, and structure of the pancreatic β-cells (SEM)	Decreases MDA, increases SOD, and improves the morphological structure of the pancreatic β-cells	[118]
	Peptides	LPS-induced RAW264.7 cells	ROS, qRT-PCR	Reduces the level of ROS; upregulates the mRNA expression of Nrf2, HO-1, and NQO1	[105]
	Peptides	SPF-grade male C57BL/6 mice	MDA and SOD	Decreases MDA and increases SOD	[1]
	1-DNJ	L929 cells, STZ-induced Kunming mice	ROS, SOD	Increases the serum SOD level in diabetic mice and reduces ROS production in glucose-induced L929 cells	[119]
Amelioration of the insulin resistance	1-DNJ	db/db mice	Intraperitoneal Glucose Tolerance Test and Intraperitoneal Insulin Tolerance Test; Western blot	Improves glucose tolerance and insulin tolerance; increases GLUT4 translocation and phosphorylation of Ser473-AKT, p85-PI3K, Tyr1361-IR-β, and Tyr612-IRS1	[120]
	1-DNJ/water extract of ML	male db/db mice	Oral Glucose Tolerance Test, and Insulin Tolerance Test; Western blot; histological analysis	Ameliorates glucose and insulin tolerance; increases IRS-1, p-PI3K, and p-Akt protein expression levels; ameliorates muscle deformation and increases muscle fiber size	[121]
	Extract	STZ-induced male Sprague-Dawley rats	Western blot; qRT-PCR; immunohistochemical staining	Increases the gene and protein expression of IRS-1, PI3K and Glut-4 in skeletal muscles	[122]
	Flavonoids	L6 skeletal muscle cells, male db/db mice, and db/m mice	Western blot; immunohistochemical staining	Upregulates the expression levels of m-GLUT4 and t-GLUT4; upregulates the expression of p-AMPK and PGC-1α	[8]
	Sangtong alkaloids	db/db mice	Serum insulin level, insulin resistance index, and insulin sensitivity index; HE staining; Western blot	Decreases serum insulin level; increases insulin resistance index and insulin sensitivity index; ameliorates histopathological damage to the pancreas; upregulates the protein expressions of P-IRS1, P-PI3K, P-AKT, and GLUT2 in liver	[123]
	Phenolics	Caco-2/insulin-resistant HepG2	qRT-PCR	Decreases mRNA expression of glucose transporters SGLT1 (0.64 ± 0.18), GLUT2 (0.14 ± 0.02), and the sucrase–isomaltas; upregulates the mRNA expressions of IRS1 (9.32-fold), Akt (17.07-fold) and GYS2 (1.5-fold); downregulates the GSK-3β (0.22-fold), PEPCK (0.49-fold) and FOXO1 (0.10-fold), and mRNA levels	[5]
	Polysaccharide	STZ-induced adult male Wistar rats	Immunofluorescence staining; qRT-PCR; Western blot	Increases the expression of IRS2, PI3Kand PKB/AKT; reduces the expression of PTP1B	[2]
Regulation of Gut microbiota	ML ethanol extract	db/db mice	16S rDNA sequencing	Alters the abundances of gut microbiota related to BAs metabolism such as *Colidextribacter*, *Muribaculum*, *Muribaculaceae*, and *Eubacterium_siraeum*_group	[124]
	1-DNJ	High-fat and STZ-induced prediabetes mice	16S rDNA sequencing; GC analysis of SCFAs	Reduces the F/B ratio to 4.71 ± 1.88; augments the abundance of S24-7, *Desulfovibrionaceae*, and *Lactobacillaceae*, and restrains the abundance of *Clostridiales* and *Lachnospiraceae*; restores total SCFA levels, but not significantly	[125]
	1-DNJ	STZ-induced diabetic mice	16S rDNA sequencing	Promotes the growth of *Lactobacillus*, *Lachnospiraceae* NK4A136 group, *Oscillibacter*, *norank Lachnospiraceae*, *Alistipes*, and *Bifidobacterium*; suppresses the growth of *Ruminococcaceae* UCG-014, *Weissella*, *Ruminococcus*, *Prevotellaceae* Ga6A1 group, *Anaerostipes*, *Klebsiella*, *Prevotellaceae* UCG-001, and *Bacteroidales* S24-7 group	[104]
	Polysaccharides	HFD-induced male C57BL/6N mice	16S rDNA sequencing	Enhances gut microbiota diversity and reduces (F/B) ratio	[32]
	Polysaccharides	HFD-induced SD male rats	16S rDNA sequencing; biochemical analysis; qRT-PCR; Western blot	Enhances the abundance of *Prevotella*, *Ruminococcus*, and *Lactobacillus*; modulates bile acid metabolism, as evidenced by reduced serum cholesterol levels; enhances mRNA expression of Cyp7a1 and Cyp8b1, and Tgr5, while suppressing Fxr mRNA expression; upregulates the protein expression of hepatic CYP7A1 and CYP8B1, and ileal TGR5, while inhibiting FXR protein levels	[33]
	Oligosaccharide	HFD-induced C57BL/6J mice	16S rDNA sequencing	Selectively promotes the growth of *Ligilactobacillus murinus*, a commensal bacterium that presented a reduced level in T2DM mice; selectively accelerates the proliferation of *L. murinus*	[34]

**Table 6:** Terms used in the search strategy.

Database	Search and Terms
Web of Science PubMed	#1. (“mulberry leaf” or “Morus alba*” or “mulberry leaves”) AND (“diabetes” or “diabetes mellitus” or “antidiabetic” or “hypoglycemi*” or “glucose regulation” or “blood glucose lowering” or “antihyperglycemic”)
Web of Science PubMed	#2. (“mulberry leaf” or “Morus alba*” or “mulberry leaves”) AND (“extract*” or “quality analysis” or “quality control” or “quality assessment” or “mechanism*”)

“*” represents any number of characters.

## Data Availability

No new data were created or analyzed in this study. Data sharing is not applicable to this article.

## Abbreviations

The following abbreviations are used in this manuscript:

IDF	International Diabetes Federation
T2DM	Type 2 diabetes mellitus
ML	Mulberry leaf
MLA	Mulberry leaf alkaloids
1-DNJ	1-deoxynojirimycin
FAG	D-fagomine
GAL-DNJ	2-*O*-α-D-galactopyranosyl deoxynojirimycin
N-Me-DNJ	*N*-Methyl-1-Deoxynojirimycin
DAB	1,4-dideoxy-1,4-imino-D-arabinitol
Glu-FAG	4-*O*-β-D-glucopyranosyl-fagomine
MLF	Mulberry leaf flavonoids
MLP	Mulberry leaf polysaccharides
Gal	Galactose
GalA	Galacturonic acid
Ara	Arabinose
Rha	Rhamnose
Glc	Glucose
GluA	Glucuronic acid
Man	Mannose
Xyl	Xylose
Fuc	Fucose
Rib	Ribose
Sor	Sorbose
MLPA	Mulberry leaf phenolic acids
MLPR	Mulberry leaf proteins
RSM	Response surface methodology
ANN	Artificial neural network
UAE	Ultrasound-assisted extraction
MAE	Microwave-assisted extraction
SFE	Supercritical fluid extraction
EAE	Enzyme-assisted extraction
PFE	High-intensity pulsed electric field extraction
DEE	Deep eutectic solvent extraction
EUCE	Enzyme-ultrasound-assisted coupling extraction
EMCE	Enzyme-microwave-assisted coupling extraction
DUCE	Deep eutectic solvent-ultrasound-assisted coupling extraction
FD	Freeze drying
SD	Spray drying
MD	Microwave drying
HBDs	Hydrogen bond donors
HBAs	Hydrogen bond acceptors
MPD	2-methyl-2,4-pentanediol
UHPLC-HRMS	Ultra-high-performance liquid chromatography–high-resolution mass spectrometry
IR	Infrared spectroscopy
^1^H-NMR	Proton nuclear magnetic resonance
GC	Gas chromatography
LC	Liquid chromatography
Q-Marker	Quality marker
Q-TOF-MS	Quadrupole time-of-flight mass spectrometry
Q-Orbitrap-MS	Q-Exactive orbitrap mass spectrometry
UHPLC-LTQ MS	Ultra-high-performance liquid chromatography coupled with linear ion trap high-resolution mass spectrometry
2D-IR	Two-dimensional infrared spectroscopy
NIR	Near-infrared
ROS	Reactive oxygen species
MAD	Malondialdehyde
SOD	Superoxide dismutas
GSH-Px	Glutathione peroxidase
PTP1B	Protein tyrosine phosphatase 1B
IRS-2	Insulin receptor substrate 2
SCFAs	Short-chain fatty acids
USBE	Ultrasonic-semi-bionic method
PSO	Particle Swarm Optimization
PRISMA 2020	Preferred Reporting Items for Systematic Reviews
TPC	Total polyphenol content
TFC	Total flavonoid content
mg GAE/g DW	Milligram of gallic acid equivalent per gram of dry weight
MLE	Mulberry leaf extract
ND	No data
